# Longitudinal risk of herpes zoster in patients with non-Hodgkin lymphoma receiving chemotherapy: A nationwide population-based study

**DOI:** 10.1038/srep14008

**Published:** 2015-09-22

**Authors:** Shih-Feng Cho, Wan-Hsuan Wu, Yi-Hsin Yang, Yi-Chang Liu, Hui-Hua Hsiao, Chao-Sung Chang

**Affiliations:** 1 Graduate Institute of Clinical Medicine, College of Medicine, Kaohsiung Medical University, Kaohsiung, Taiwan; 2 Division of Hematology & Oncology, Department of Internal Medicine, Kaohsiung Medical University Hospital, Kaohsiung Medical University, Kaohsiung, Taiwan; 3 School of Medicine, I-Shou University, Kaohsiung, Taiwan; 4 School of Pharmacy, Kaohsiung Medical University, Kaohsiung, Taiwan; 5 Division of Hematology and Oncology, E-Da Hospital, Kaohsiung, Taiwan

## Abstract

This study investigated the incidence of and risk factors for herpes zoster in patients with non-Hodgkin lymphoma (NHL) who were receiving anti-lymphoma treatment. The overall incidence density of herpes zoster was 12.21% (472/3865); 11.79% (258/2188) of the patients received conventional chemotherapy and 12.76% (214/1677) of the patients received rituximab-containing chemotherapy. For the patients who received conventional chemotherapy, the risk factors included female gender, multiple courses of chemotherapy and autologous hematopoietic stem cell transplantation. For the patients who received rituximab-containing chemotherapy, the risk factors included female gender, diabetes mellitus, multiple courses of chemotherapy, autologous hematopoietic stem cell transplantation and higher accumulated rituximab dose. The majority of the herpes zoster episodes occurred within the first two years after the diagnosis of NHL. After adjusting for the propensity score matching, rituximab-containing chemotherapy was not associated with a higher overall incidence density of herpes zoster (*P* = 0.155). However, the addition of rituximab to conventional chemotherapy increased the short-term risk of herpes zoster with adjusted odd ratios of 1.38 (95% confidence intervals (CI) = 1.05–1.81, *P* = 0.021) and 1.37 (95% CI = 1.08–1.73, *P* = 0.010) during the 1-year and 2-year follow-up periods, respectively.

Non-Hodgkin lymphoma (NHL) is the most common hematologic malignancy and includes various histological subtypes of lymphoma that develop from lymphatic system. In recent decades, the long-term outcome of patients with NHL has significantly improved. One possible cause for this improvement is the advance in therapeutic agent development, which has improved the efficiency of chemotherapy. The introduction of the anti-CD20 monoclonal antibody rituximab was an important breakthrough for patients with B-cell NHLs that express the CD20 antigen. The incorporation of rituximab into conventional chemotherapy has improved the clinical outcome of patients with diffuse large B-cell lymphoma and follicular lymphoma significantly^1^,^2^,^3^,^4^,^5^. Because the outcomes of patients with NHL have improved, the short- and long-term effects of treatment-related adverse events on the quality of life might emerge as issues. Previous researches have revealed that anti-lymphoma chemotherapy is associated with the reactivation of various viral infections such as hepatitis B, cytomegalovirus and varicella-zoster virus^6^,^7^.

Herpes zoster (HZ) is viral disease that is characterized by painful vesicular skin lesions that are limited to the dermatomal regions. The pathophysiology of herpes zoster is associated with the reactivation of varicella-zoster virus in people who have previously been infected. The incidence of herpes zoster is between 2.15 and 4.97 cases per 1000 person-years in the general population^8^,^9^,^10^,^11^, and the incidence and severity of symptoms increases with age^8^,^9^. The reactivation of varicella-zoster virus is associated with compromised immunity due to conditions such as diabetes mellitus^12^, autoimmune diseases^13^,^14^, human immunodeficiency virus (HIV) infection^15^ and receipt of a transplant^16^,^17^,^18^. The incidence rate and severity of herpes zoster were also higher among the patients with malignancies and following cancer-related therapy^19^,^20^. The patients who recovered from herpes zoster often suffered from various neurologic consequences such as neuralgia, cranial nerve palsy, polyneuritis, myelitis, meningitis or encephalitis^21^. Post-herpetic neuralgia is the most common neurologic complication, persists for several months, and can be difficult to manage. For patients with NHL, herpes zoster and its complications can affect the quality of life and delay scheduled anti-lymphoma treatment^22^,^23^,^24^. In the rituximab era, the combination of rituximab and conventional chemotherapy might further suppress immune function and increase the risk of herpes zoster. However, studies that are relevant to herpes zoster in NHL patients after anti-lymphoma therapy are relatively rare.

We conducted this nationwide population-based study to elucidate the incidence of and risk factors for herpes zoster in patients receiving anti-lymphoma treatment. Additionally, we investigated the effect of the addition of rituximab to conventional chemotherapy on the incidence of herpes zoster.

## Material and Methods

### National Health Insurance Research Database

The data for this study were from the National Health Insurance Research Database (NHIRD), which is derived from the National Health Insurance (NHI) of Taiwan and has been which was implemented since March 1st of 1995. This nationwide population-based database provided the most comprehensive information for this study.

For patients with certain severe illnesses, such as autoimmune diseases, end-stage renal disease and malignant diseases, the NHI has established a registration system (Registry for Catastrophic Illness Patients). The patients who met the criteria for catastrophic illness are registered, and their responsibilities for all co-payments are waived. For patients with NHL, certificates of catastrophic illness are issued when the lymphoma pathologically proven.

### Inclusion and exclusion criteria for the study population

We enrolled patients with NHL who were aged 20 years and older. The patients with NHL were defined by the International Classification of Disease, 9th Revision, Clinical Modification (ICD-9-CM) codes for NHL (200.0–200.8 and 202.0–202.9) and then validated based on Registry for Catastrophic Illness Patients. The patients with herpes zoster (ICD-9 code: 053.0–053.9) were defined based on having at least one service claim for inpatient or outpatient care. The exclusion criteria were the following: patients with previous or coexisting malignant diseases and patients with a diagnosis of another malignancy within 6 months of the NHL diagnosis.

Our study analyzed the incidence of herpes zoster in patients receiving anti-lymphoma therapy. In Taiwan, the conventional first-line chemotherapy regimens include cyclophosphamide, doxorubicin, vincristine and prednisolone (CHOP) and cyclophosphamide, epirubicin, vincristine and prednisolone (CEOP). For patients with B-cell lymphomas, including diffuse large B-cell lymphoma or follicular lymphoma, rituximab (R) was added to conventional chemotherapy (i.e., R-CHOP or R-CEOP) after it was introduced and approved by the NHI in 2002. We identified only two groups for our analysis. For the patients who underwent first-line conventional chemotherapy, the regimen of chemotherapy was required to be CHOP or CEOP. For the patients who underwent rituximab-containing chemotherapy, the regimen of chemotherapy was required to R-CHOP or R-CEOP. To evaluate the dose-dependent of rituximab, we divided the patients who were receiving rituximab-containing chemotherapy into groups with higher (≥3501 mg) and lower accumulated doses (≤3500 mg). This cut-off value was the approximate median accumulated rituximab dose among the subjects.

**Figure 1:**
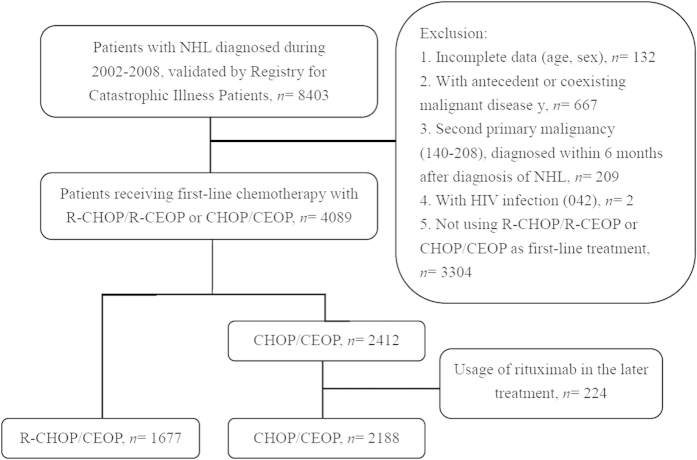
The flow chart summarizing the enrollment of subjects for this study from National Health Insurance Research Database of Taiwan.

We also identified a subgroup of patients who had undergone multiple courses of chemotherapy that were suggestive of clinical statuses of relapsed or refractory NHL. Patients who had undergone chemotherapy after the first-line R-CHOP/R-CEOP or CHOP/CEOP chemotherapy were enrolled. The patients who had undergone autologous hematopoietic stem cell transplantation were also enrolled for this analysis. The duration of this study was from 2002 to 2008. The detailed flow chart of the patients who met the inclusion or exclusion criteria for the study population was in shown in the Figure 1. And the specific number of patients enrolled in each year of 2002–2008 was summarized in Supplementary Table S1. All of the enrolled NHL patients were tracked until death or the end of 2008.

Regarding the analyses of the comorbidities, we used the Charlson comorbidity index (CCI), which was defined by Charlson *et al.*, and the Deyo-Charlson comorbidity index; these indices were based on ICD-9 codes in claims data. The CCI was calculated to define the existing comorbidities using all of the diagnostic codes for the year prior to the index date for all of the inpatients and outpatients. This index has been widely used in analyses of the effects of comorbidity on mortality^25^,^26^,^27^. Some parameters were identified for subgroup analysis, including diabetes mellitus (ICD-9 code: 250), hepatitis B infection (ICD-9 codes: 070.2 and 070.3; V02.61), hepatitis C infection (ICD-9 codes: 070.41, 070.44, 070.51, and 070.54; V02.62), HIV infection (ICD-9 code: 042), systemic lupus erythematosus (ICD-9 code: 710) and rheumatoid arthritis (ICD-9 code: 714).

### Statistics

The frequencies of each categorical variable were compared with chi-square tests (χ2 test) or Fisher’s exact tests. Propensity score matching was performed to correct for sample selection bias. Relative risk analyses were performed by calculating the odds ratios (ORs) and 95% confidence intervals (CIs) via multivariate logistic regression.

All statistical analyses were based on two-sided hypothesis tests with a significance level of *P* < 0.05. The analyses were performed using SPSS version 17.0 (SPSS, Chicago, IL, USA).

### Ethical Approval

The NHIRD is totally de-identified and encrypted database for research purposes only. This study was conducted in accordance with the Helsinki Declaration and was also reviewed and approved by Institutional Review Board of Kaohsiung Medical University Hospital (KMUH-IRB-EXEMPT-20140027).

## Results

From 2002 to 2008, there were 3865 NHL patients with median age of 55 years who received CHOP/CEOP or R-CHOP/R-CEOP as front-line treatments; 2188 of these patients (median age: 52 years) received CHOP/CEOP, and 1677 patients (median age: 59 years) received R-CHOP/R-CEOP. With follow-ups of 5853 and 3090 person-years, herpes zoster was noted in 258 (11.79%) and 214 (12.76%) patients in the CHOP/CEOP and R-CHOP/R-CEOP groups, respectively. The overall incidence density was 12.21%. In the CHOP/CEOP group, higher rates of herpes zoster were associated with following factors: female gender, age below 50 years, multiple courses of chemotherapy, and autologous hematopoietic stem cell transplantation. In the R-CHOP/R-CEOP group, higher rates of herpes zoster were associated with the following factors: female gender, age between 51 and 64 years, diabetes mellitus, multiple courses of chemotherapy, autologous hematopoietic stem cell transplantation, and higher accumulated rituximab doses (≥3501 mg). The general characteristics of the NHL patients with and without herpes zoster are summarized in Table 1.

Multivariate analyses were also performed to investigate the risk factors for herpes zoster. In the CHOP/CEOP group, female gender, multiple courses of chemotherapy and autologous hematopoietic stem cell transplantation were associated with greater risks for herpes zoster. The elder patients (≥65 years) exhibited a lower risk of herpes zoster. In the R-CHOP/R-CEOP group, the risk factors for herpes zoster included female gender, diabetes mellitus, higher rituximab dosage (≥3501 mg), multiple courses of chemotherapy and autologous hematopoietic stem cell transplantation (Table 2).

**Figure 2:**
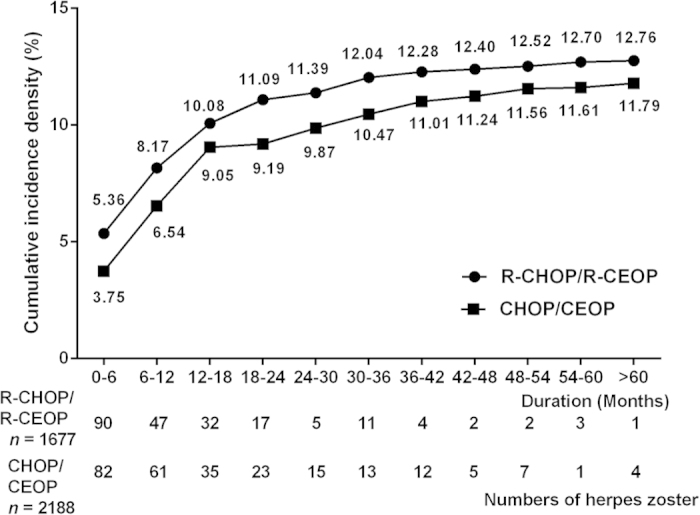
The annual case numbers and cumulative incidence density of herpes zoster in patients with NHL who received R-CHOP/R-CEOP or CHOP/CEOP.

The annual incidences of herpes zoster were also evaluated in these two groups. The onset of herpes zoster primarily occurred in the first two years after diagnoses of NHL; 186 patients (86.92%) in the R-CHOP/R-CEOP group and 201 patients (77.91%) in the CHOP/CEOP group fit this pattern (Fig. 2). Additionally, we identified the patients in whom herpes zoster occurred between courses of chemotherapy; 163 (63.18%) cases and 158 (73.83%) cases in the CHOP/CEOP and R-CHOP/R-CEOP groups, respectively, fit this pattern.

**Figure 3:**
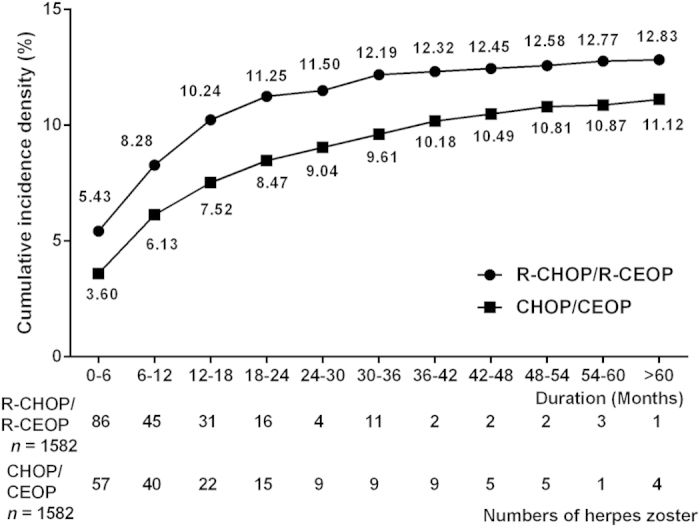
The annual case numbers and cumulative incidence density of herpes zoster in patients with NHL who received R-CHOP/R-CEOP or CHOP/CEOP after propensity score matching.

The effect of rituximab on the incidence of herpes zoster was also investigated. We created two groups with CCI scores of 0 and ≥1. After adjusting for age, gender and CCI via propensity score matching, there were 1582 patients in each group. 203 patients (12.83%) in the R-CHOP/R-CEOP group and 176 patients (11.12%) in CHOP/CEOP group developed herpes zoster. There was no significant difference in the overall incidences between these two groups (*P* = 0.155; Table 3). Analysis of the annual incidence of herpes zoster revealed that the incidence rate also reached a plateau in the first two years after diagnosis of NHL with 178 patients (87.68%) in the R-CHOP/R-CEOP group and 134 patients (76.14%) in the CHOP/CEOP group (Fig. 3).

Because the majority of the cases of herpes zoster developed within the first two years, we further investigated whether rituximab-containing chemotherapy increased the risk of herpes zoster within this period. Multivariate analysis revealed that the R-CHOP/R-CEOP group exhibited a significantly greater risk of herpes zoster within the 1-year (adjusted OR: 1.38, 95% CI = 1.05–1.81, *P* = 0.021) and 2-year follow-up periods (adjusted OR: 1.37, 95% CI = 1.08–1.73, *P* = 0.010). After 7 years of follow-up, there were no significant differences between these two groups (adjusted OR: 1.17, 95% CI = 0.95–1.46, *P* = 0.148; Table 4).

## Discussion

In the present study, we analyzed the incidences of herpes zoster among patients with NHL who underwent conventional or rituximab-containing chemotherapy. The overall incidences of both groups were higher than those of the general population, which might have been due to disease- or treatment-related factors. Additionally, the majority of herpes zoster cases developed within the first two years of the diagnosis of NHL, and this observation might be attributable to lymphoma-related treatment.

The present study investigated the risk factors for herpes zoster in patients who underwent conventional or rituximab-containing chemotherapy. We observed that female patients exhibited a higher incidence. This result is concordant with those of some previous studies, although the detailed mechanism responsible for this result is not clear^28^,^29^,^30^. The patients who received multiple courses of chemotherapy or autologous hematopoietic stem cell transplantation also exhibited significantly elevated risks for herpes zoster. The possible cause of this observation might be related to impaired immunity. Among the patients who received multiple courses of chemotherapy, uncontrolled underlying disease was found to be related to immunocompromised status, and prolonged chemotherapy courses might further suppress immune function. Regarding the patients who had received autologous hematopoietic stem cell transplantation, potent conditioning chemotherapy prior to transplantation might also have suppressed their immune function and hence increased the risk for herpes zoster. Among the patients who had received rituximab-containing chemotherapy, two additional risk factors, i.e., diabetes mellitus and a greater accumulated dose of rituximab, were identified. The cause of the significantly elevated risk for herpes zoster in patients with diabetes mellitus in the R-CHOP/R-CEOP group but not in the conventional chemotherapy group is not clear. In the patients with greater accumulated rituximab doses, the greater risk of herpes zoster might have been due to a stronger immunosuppressant effect. Based on above results, the development of herpes zoster was closely related to impaired immune function.

Our present study also revealed that the incorporation of rituximab into conventional chemotherapy significantly increased the risk of herpes zoster within the first 2 years after NHL diagnosis. However, there was no significant difference in the overall incidences between the two groups after the 7-year follow-up period. Moreover, the cumulative incidence patterns were similar in both groups; i.e., the majorities of the herpes zoster episodes occurred within the first 2 years. A previous study revealed that rituximab administration causes B-cell depletion and affects cellular and humoral immunities, which might explain the greater risk of herpes zoster among the patients who had received rituximab-containing chemotherapies^31^. We made some observations that partially support the notion that rituximab administration was associated with an increase in the short-term but not the long-term risk of herpes zoster. First, rituximab administration can lead to the rapid depletion of CD20 expression by B cells due to antibody-dependent cell-mediated cytotoxicity^32^,^33^. However, CD20 is a surface antigen of mature B cells and the most of malignant B cells rather than the primitive cells, such as progenitor B cells and precursor B cells, during B lymphocyte differentiation. Second, a previous study revealed that normal B-cell numbers and functions recover gradually at approximately six to nine months after the discontinuation of rituximab^34^. Based on this finding, the effects of rituximab on the immune system are relatively short lived, and after prolonged duration follow-up, the incidence of herpes zoster among the patients who took rituximab were similar to those of the patients who received conventional chemotherapy.

The present study based on the NHIRD has several strengths. First, this nationwide database covers a large number of patients and is free of selection bias. The study cohort is representative of the general Taiwanese population. Second, this large-scale database is comprehensive and unlikely to be missing any substantial amount of data regarding medical procedures and drug administrations. However, some limitations should be noted. First, detailed clinical data, including definite histological subtypes, are not unavailable in this database. Thus, whether different histological subtypes affected the incidence of herpes zoster is unclear. Second, the patients with herpes zoster were defined based on the ICD-9-CM codes. This method might be less accurate. However, the Bureau of the NHI in Taiwan periodically reviews the medical records to confirm the accuracy of the diagnoses and the quality of care by randomly sampling a certain percentage of the claims from every hospital. It is generally believed that the quality and accuracy of disease coding in the NHIRD are acceptable for epidemiological analyses. Third, we might have lost some patients with herpes zoster if they received chemotherapy regimens other than the standard R-CHOP/R-CEOP or CHOP/CEOP. Fourth, clinical data prior to 1995 was not available because the NHI of Taiwan was not implemented before this time. Hence, we were unable to separately analyze the subgroup of patients who had experienced previous episodes of herpes zoster. Fifth, the follow-up duration for patients enrolled in 2007 and 2008 is relatively short. However, the majority of the herpes zoster episodes were noted within the first two years after the diagnosis of NHL. Based on the above result, longer duration of follow-up may get similar result in this subgroup. Finally, some patients with mild herpes zoster symptoms likely did not seek medical advice, which might have resulted in the underestimation of the incidence of herpes zoster.

In summary, this study evaluated the incidence of and risk factors for herpes zoster in NHL patients who had received anti-lymphoma therapy. Because the overall incidence of herpes zoster was higher among the patients with NHL during chemotherapy, prophylaxes against herpes zoster might be considered, particularly for patients with the risk factors. Additionally, the incorporation of rituximab into conventional chemotherapy was associated with an increased short-term risk of herpes zoster.

## Additional Information

**How to cite this article**: Cho, S.-F. *et al.* Longitudinal risk of herpes zoster in patients with non-Hodgkin lymphoma receiving chemotherapy: A nationwide population-based study. *Sci. Rep.* **5**, 14008; doi: 10.1038/srep14008 (2015).

## Supplementary Material

Supplemantary Table S1

## Figures and Tables

**Table 1:** General characteristics of the study population with and without herpes zoster.

	CHOP/CEOP(*n*=2188)			R-CHOP/R-CEOP(*n*=1677)		
	HZ (+)*n* = 258	HZ (−)*n* = 1930	*P-value*	HZ (+)*n *= 214	HZ (−)*n *= 1463	*P-value*
**Gender**			0.001			0.023
Male (*n*, %)	128(9.86)	1169(91.14)		101(11.06)	812(88.94)	
Female (*n*, %)	130(14.59)	761(85.41)		113(14.79)	651(85.21)	
**Age (years)**			0.020			0.012
≤50 (*n*, %)	136(13.22)	893(86.78)		62(11.99)	455(88.01)	
51–64 (*n*, %)	75(12.34)	533(87.66)		88(16.12)	458(83.88)	
≥65 (*n*, %)	47(8.53)	504(91.47)		64(10.42)	550(89.58)	
**Hepatitis B**			0.297			0.266
With (*n*, %)	24(14.29)	144(85.71)		26(15.48)	142(84.52)	
Without (*n*, %)	234(11.58)	1786(88.42)		188(12.46)	1321(87.54)	
**Hepatitis C**			0.956			0.929
With (*n*, %)	16(11.94)	118(88.06)		15(12.5)	105(87.5)	
Without (*n*, %)	242(11.78)	1812(88.22)		199(12.78)	1358(87.22)	
**DM**			0.885			0.031
With (*n*, %)	14(11.38)	109(88.62)		21(19.44)	87(80.56)	
Without (*n*, %)	244(11.82)	1821(88.18)		193(12.30)	1376(87.70)	
HIV*			1.000			1.000
With (*n*, %)	1(9.10)	10(90.90)		0(0)	4(100)	
Without (*n*, %)	257(11.81)	1920(88.19)		214(12.79)	1459(87.21)	
SLE*			0.607			0.560
With (*n*, %)	0(0.0)	8(100)		1(16.67)	5(83.33)	
Without (*n*, %)	258(11.83)	1922(88.17)		213(12.74)	1458(87.26)	
RA*			0.610			0.125
With (*n*, %)	0(0.0)	9(100)		3(30)	7(70)	
Without (*n*, %)	258(11.84)	1921(88.16)		211(12.66)	1456(87.34)	
**Auto-HSCT**			<0.001			<0.001
With (*n*, %)	27(39.13)	42(60.87)		13(30.95)	29(69.05)	
Without (*n*, %)	231(10.90)	1888(89.10)		201(12.29)	1434(87.71)	
**Multiple C/T**			<0.001			<0.001
With (*n*, %)	118(16.03)	618(83.97)		79(19.17)	333(80.83)	
Without (*n*, %)	140(9.64)	1312(90.36)		135(10.67)	1130(89.33)	
**R dose (mg)**						0.001
≤3500 (*n*, %)				75(9.81)	689(90.19)	
≥3501 (*n*, %)				139(15.22)	774(84.78)	

HZ, herpes zoster; DM, diabetes mellitus; HIV, Human immunodeficiency virus; SLE, systemic lupus erythematosus; RA, rheumatoid arthritis; Auto-HSCT, autologous hematopoietic stem cell transplantation; Multiple C/T, multiple courses of chemotherapy; R, rituximab.

^*^Analysis by Fisher’s exact test.

**Table 2:** Multivariate logistic regression analysis to investigate the risk factors for herpes zoster in the patients who received R-CHOP/R-CEOP and CHOP/CEOP.

		CHOP/CEOP (*n*=2188)			R-CHOP/R-CEOP (*n*=1677)		
		*n*	OR (95% CI)	*P-value*	*n*	OR (95% CI)	*P-value*
**Gender**	Male (ref.)	1297	1		913	1	
	Female	891	1.53(1.18–1.99)	0.001	764	1.44(1.08–1.93)	0.014
**Age (years)**	≤50 (ref.)	1029	1		517	1	
	51–64	608	0.92(0.68–1.25)	0.595	546	1.38(0.97–1.98)	0.076
	≥65	551	0.63(0.44–0.89)	0.009	614	0.93(0.63–1.36)	0.699
**Multiple C/T**	No (ref.)	1452	1		1265	1	
	Yes	736	1.50(1.13–1.99)	0.005	412	1.73(1.25–2.38)	0.001
**Auto-HSCT**	No (ref.)	2119	1		1635	1	
	Yes	69	3.67 (2.14–6.28)	<0.001	42	2.15(1.05–4.41)	0.037
**DM**	No (ref.)				1569	1	
	Yes				108	1.78(1.06–2.99)	0.031
**R dose(mg)**	≤3500 (ref.)				764	1	
	≥3501				913	1.58(1.17–2.14)	0.003

OR, odds ratio; 95%CI, 95% confidence interval; CHOP, cyclophosphamide, doxorubicin, vincristine, prednisolone; CEOP, cyclophosphamide, epirubicin, vincristine, prednisolone; Multiple C/T, multiple courses of chemotherapy; Auto-HSCT, autologous hematopoietic stem cell transplantation; DM, diabetes mellitus; R, rituximab.

**Table 3:** General characteristics of the study cohort and incidence of herpes zoster before and after propensity score matching.

		Before matching						*P-value*	After matching						*P-value*
		R-CHOP/R-CEOP(*n *= 1,677)			CHOP/CEOP (*n *= 2,188)				R-CHOP/R-CEOP(*n *= 1,582)			CHOP/CEOP (*n *= 1,582)			
		*n*	Person-YearsofFollow-Up	%	*n*	Person-YearsofFollow-Up	%		*n*	Person-YearsofFollow-Up	%	*n*	Person-YearsofFollow-Up	%	
Gender	Female	764	1409	45.6	891	2571	40.7	0.003	685	1270	43.3	679	1926	42.9	0.858
	Male	913	1681	54.4	1,297	3282	59.3		897	1671	56.7	903	2167	57.1	
Age	≤50	517	915	30.8	1,029	3168	47.0	<0.001	517	915	32.7	517	1664	32.7	0.967
(years)	51–64	546	1021	32.6	608	1556	27.8		520	1000	32.9	514	1300	32.5	
	≥65	614	1155	36.6	551	1129	25.2		545	1026	34.5	551	1129	34.8	
CCI	0	1,613	3044	96.2	2,145	5777	98.0	0.001	1,547	2916	97.8	1,547	4028	97.8	1.000
	≥1	64	46	3.8	43	76	2.0		35	25	2.2	35	64	2.2	
HZ	No	1,463	2523	87.2	1,930	4887	88.2	0.373	1,379	2401	87.2	1,406	3454	88.9	0.155
	Yes	214	567	12.8	258	967	11.8		203	540	12.8	176	639	11.1	

R, rituximab; CHOP, cyclophosphamide, doxorubicin, vincristine, prednisolone; CEOP, cyclophosphamide, epirubicin, vincristine, prednisolone; CCI, Charlson Comorbidity Index .

**Table 4:** Odds ratios (ORs) and 95% confidence intervals (CIs) of herpes zoster (HZ) among the patients who received R-CHOP/R-CEOP or CHOP/CEOP during the 1-, 2-, and 7-year follow-up periods.

	1-year follow-up period		2-year follow-up period		7-year follow-up period	
HZ	R-CHOP/R-CEOP	CHOP/CEOP	R-CHOP/R-CEOP	CHOP/CEOP	R-CHOP/R-CEOP	CHOP/CEOP
Yes (*n*, %)	131(8.28)	97 (6.13)	178(11.25)	134(8.47)	203(12.83)	176(11.12)
No (*n*, %)	1451(91.72)	1485(93.87)	1404(88.75)	1448(91.53)	1379(87.17)	1406(88.88)
Crude OR (95% CI)	1.38^a^(1.05–1.82)	1	1.37^c^(1.08–1.73)	1	1.18^e^(0.95–1.46)	1
Adjust OR (95% CI)	1.38^b^(1.05–1.81)	1	1.37^d^(1.08–1.73)	1	1.17^f^(0.95–1.46)	1

*Adjustments were made for the patients’ genders, ages and Charlson comorbidity indices.

(*P-values*: a: 0.020; b: 0.021; c: 0.009; d: 0.010; e: 0.140; and f: 0.148).

R, rituximab; CHOP, cyclophosphamide, doxorubicin, vincristine, prednisolone; CEOP, cyclophosphamide, epirubicin, vincristine, prednisolone.
