# Supplementary material for: Longitudinal risk of herpes zoster in patients with non-Hodgkin lymphoma receiving chemotherapy: A nationwide population-based study
Source: Sci Rep. 2015 Sep 22;5:14008. doi: 10.1038/srep14008 (PMC4585724; doi:10.1038/srep14008)
Supplement: Supplemantary Table S1 [file srep14008-s1.pdf]

SUPPORTING MATERIAL

Longitudinal risk of herpes zoster in patients with non-Hodgkin lymphoma receiving chemotherapy: A nationwide population-based study

Shih-Feng Cho[1, 2], Wan-Hsuan Wu[3], Yi-Hsin Yang[4], Yi-Chang Liu[2], Hui-Hua Hsiao[2], Chao-Sung Chang[3, 5*]

1. Graduate Institute of Clinical Medicine, College of Medicine, Kaohsiung Medical University, Kaohsiung, Taiwan

2. Division of Hematology & Oncology, Department of Internal Medicine, Kaohsiung Medical University Hospital, Kaohsiung Medical University, Kaohsiung, Taiwan

3. School of Medicine, I-Shou University, Kaohsiung, Taiwan

4. School of Pharmacy, Kaohsiung Medical University, Kaohsiung, Taiwan

5. Division of Hematology and Oncology, E-Da Hospital, Kaohsiung, Taiwan

Correspondence to: Prof. Chao-Sung Chang*

Affiliation: Division of Hematology and Oncology, E-Da Hospital, Kaohsiung, Taiwan

Address: No.1, Yida Road, Jiaosu Village, Yanchao District, Kaohsiung City 82445, Taiwan

Tel: +886 76150011

Fax: +886 76150940

E-mail: ccschang@gmail.com

Material and Methods

Supplementary Table S1. Specific number of patients enrolled in each year of 2002-2008.

Year	All NHL patients (*n*= 3865)	R-CHOP/R-CEOP (*n*= 1677)	CHOP/CEOP (*n*= 2188)
2002	548	52	496
2003	489	81	408
2004	522	164	358
2005	509	195	314
2006	579	363	216
2007	605	431	174
2008	613	391	222

Note: There were 129 patients with follow-up of 7 years, including 7 in the R-CHOP/R-CEOP group and 122 in the CHOP/CEOP group.
